# Endometrial receptivity characteristics in patients with repeated implantation failure: a study using LASSO regression and Bayesian generalized linear model analysis

**DOI:** 10.3389/fmed.2026.1804397

**Published:** 2026-06-08

**Authors:** Panpan Zhao, Yuexin Yu

**Affiliations:** Department of Reproductive Medicine, General Hospital of Northern Theater Command, Shenyang, China

**Keywords:** Bayesian generalized linear modeling, endometrial receptivity, LASSO regression, repeated implantation failure, ultrasound assessment

## Abstract

**Background:**

Repeated implantation failure (RIF) is a key challenge in assisted reproductive technology (ART), and its mechanism is closely related to Endometrial receptivity (ER). Although the effects of some Endometrial receptivity parameters on pregnancy outcomes have been investigated, there is a lack of standardized assessment of the interaction of multidimensional parameters and systematic prediction models.

**Objective:**

This study aimed to investigate the key factors influencing Endometrial receptivity in patients with RIF using LASSO regression and Bayesian generalized linear models, with an emphasis on clinical interpretability and uncertainty quantification.

**Methods:**

This was a retrospective cohort study. A total of 506 women patients who underwent frozen–thawed embryo transfer (FET) at the Department of Reproductive Medicine from January 2023 to December 2024 were enrolled. All patient data and examination results were retrospectively collected from the electronic medical records system of our hospital. They were divided into a case group (RIF group, *n* = 147) and a control group (control group, *n* = 359) based on their medical histories. Eight Endometrial receptivity parameters were assessed via ultrasound. Variable selection was performed using LASSO regressionstrictly within the training set combined with Bayesian modeling to quantify parameter effects.

**Results:**

The LASSO regression identified endometrial blood flow branches, endometrial arterial resistance index (RI), endometrial arterial pulsatility index (PI), endometrial arterial peak systolic velocity/end-diastolic velocity ratio (S/D), and endometrial peristaltic frequency as the most predictive variables. The Bayesian model indicated that increased endometrial blood flow branch count reduced the risk of RIF (OR = 0.1, 95% CI:0.06–0.17). Elevated endometrial arterial RI (OR = 1.21, 95% CI: 1.04–1.44) increased RIF risk, while decreased endometrial arterial PI (OR = 1.48, 95% CI: 1.06–2.10) showed an inverse association with RIF. Higher endometrial arterial peak systolic velocity/end-diastolic velocity ratio (OR = 3.63, 95% CI: 1.54–9.03) was positively correlated with RIF risk. Additionally, increased endometrial peristaltic frequency (OR = 1.93, 95% CI: 1.16–3.17) significantly elevated RIF risk. 10-fold cross-validation on the training set yielded a mean AUC of 0.904 ± 0.021. The model demonstrated excellent discriminatory ability (AUC = 0.911) and calibration performance (C-statistic = 0.94). Standardized net benefit analysis confirmed the clinical utility of the model across a wide range of risk thresholds (1:100 to 100:1).

**Conclusion:**

This study is the first to combine LASSO and Bayesian methods to construct a predictive model for RIF, highlighting the critical role of endometrial hemodynamic and peristaltic characteristics. The Bayesian framework offers uncertainty estimates that can guide personalized interventions. These findings provide new targets for precision diagnosis and treatment.

## 1 Background

The widespread use of assisted reproductive technology (ART) has led to a significant increase in pregnancy rates among infertile patients. However, approximately 10% of patients still fail to achieve a pregnancy due to repeated implantation failure (RIF) (1). RIF is defined as failure to reach a clinical pregnancy in an adult woman under 40 years of age after transfer of at least 3 good quality embryos in 3 fresh or frozen cycles, where good quality embryos include Day 3 embryos (according to the Veeck classification, morphologically good cleavage-stage embryos are defined as those with a cell number ≥ 8, uniform blastomere size, and a fragmentation rate < 10%.) and blastocysts (according to the Gardner score (2). Well-formed blastocysts were defined as Day 5 with a score of 3BB and above). It has been estimated that reduced endometrial compatibility accounts for approximately two-thirds of the many factors that contribute to embryo implantation failure (1). However, the assessment of Endometrial receptivity still faces many challenges: first, Endometrial receptivity is a dynamic, multifactorial, and complex process, which involves multidimensional indicators such as morphology, hemodynamics, and molecular biology; second, the existing studies focus on a single parameter, such as the endometrial thickness or the blood flow index, and lack a systematic analysis of the interactions of multiple parameters; and third, the problems of downscaling of high-dimensional data and quantification of the uncertainty of the model have not yet been adequately addressed. Recent advances in machine learning have greatly enhanced clinical prediction modeling, including multi-modal data fusion, robust feature selection techniques, Bayesian deep learning for uncertainty quantification, and interpretable clinical prediction models. To address the aforementioned challenges, this study innovatively integrated LASSO regression (Least Absolute Shrinkage and Selection Operator) with Bayesian generalized linear models. Unlike conventional stepwise regression, our approach provides not only point estimates but also full posterior distributions (credible intervals) for each predictor, enabling clinicians to assess the uncertainty of risk factors. The contribution of this study lies in translating a standard modeling pipeline into a clinically actionable tool for RIF, rather than claiming methodological novelty.

## 2 Methods/experimental

### 2.1 Research subjects

#### 2.1.1 Selection criteria

##### 2.1.1.1 Inclusion criteria

(1) Women patients who underwent Frozen–thawed embryo transfer (FET) for assisted conception and embryo transfer in our department;
(2) Age < 40 years old;

##### 2.1.1.2 Exclusion criteria

(1) Intermural and/or subplasma fibroids > 4 cm that press into the uterine cavity and may distort the uterine cavity;
(2) Stage III-IV endometriosis;
(3) Thin endometrium (< 6 mm);
(4) Chromosomal abnormalities (either partner or embryos);
(5) Autoimmune diseases (e.g., antiphospholipid syndrome, systemic lupus erythematosus);
(6) Thyroid dysfunction (TSH > 4.0 mIU/L or < 0.27 mIU/L);
(7) History of intrauterine adhesions;
(8) Embryo quality below 3BB (blastocyst) or grade I 8-cell (cleavage-stage) in the current transfer cycle.

#### 2.1.2 Grouping criteria

Of all the patients enrolled in the study, those who failed to achieve a clinical pregnancy after the transfer of at least 3 high-quality embryos in 3 transfer cycles were included in the RIF group; those who achieved a clinical pregnancy in the first transfer cycle were included in the control group.

According to the inclusion–exclusion criteria and grouping criteria, a total of 506 patients were included in this study, including 147 patients in the RIF group and 359 patients in the control group. All patients were divided into two groups, the training set and the validation set, according to the ratio of 7: 3. There were 103 cases in the RIF group and 251 cases in the control group in the training set, and 44 cases in the RIF group and 108 cases in the control group in the validation set.

### 2.2 Research methodology

#### 2.2.1 Basic information collection

(1) Basic information mainly included the patient’s name, age, body mass index (BMI), type of infertility, causes of infertility, and years of infertility;
(2) The primary test data include anti-Müllerian duct hormone (AMH), basal estrogen (E2), basal progesterone (P), basal follicular stimulating hormone (FSH), basal luteinizing hormone (LH). We also recorded the number of previous embryo transfers and the embryo stage (cleavage-stage/blastocyst) in the current transfer cycle.

#### 2.2.2 Endometrial preparation program

On days 2 to 5 of the menstrual cycle, patients were started on oral Femoston (Complex Packing Estradiol Tablets/Estradiol and Dydrogesterone Tablets) at an initial dose of 4 mg/day for 8 days. After 8 days of treatment, endometrial thickness was measured by vaginal ultrasound, followed by blood sampling for serum E_2_ levels. Given the individual differences between patients, a customized dosing regimen was be developed for each patient based on their specific condition. When the endometrial thickness reached 6 mm or more, and no dominant follicles were detected on examination, endometrial transformation was initiated: 40 mg of progesterone was given intramuscularly every day, while 400 mg of progesterone soft gels were taken orally every day.

The minimum threshold of 6 mm for initiating luteal transformation was selected based on the following evidence:

(1) The Expert Consensus on Clinical Diagnosis and Treatment of Repeated Implantation Failure issued by the Chinese Medical Doctor Association clearly (1) states that the clinical pregnancy rate is significantly reduced when endometrial thickness is < 6 mm, and cancelation of the transfer cycle is recommended;
(2) Excluding patients with thin endometrium avoids its interference with RIF risk assessment, allowing us to more accurately investigate the independent effects of endometrial hemodynamics and peristaltic function on implantation failure.

The exact timing of the frozen–thawed embryo transfer (FET) depended on the type of thawed embryo: for day 3 cleavage stage embryos, the transfer was scheduled on days 3–4 after endometrial transformation; for day 5 blastocysts, the transfer was scheduled on days 5–6 after endometrial transformation.

#### 2.2.3 Ultrasound assessment of endometrial receptivity

On the day of transplantation, two groups of patients were scheduled to undergo transvaginal ultrasonography as a means of evaluating eight indicators of Endometrial receptivity. The equipment used was a Voluson E8 color ultrasound machine from GE, USA, with a transvaginal probe set at 5–9 MHz, and the entire ultrasound examination was performed by the same imaging specialist, following the same standardized protocol. For each indicator, three consecutive measurements were taken, and the average of the three measurements was recorded.

Specifics included:

(1) Endometrial thickness: A standardized image of a longitudinal section of the endometrium was obtained, and the endometrial thickness was measured at a distance of 1.5–2.0 cm from the uterine fundus.
(2) Endometrial morphology: endometrial typing is the different echogenic reflections of the endometrium and the adjacent myometrium. Gonen’s typing (3) divides the endometrium into 3 types: type A, in which the endometrium shows a clear trilinear sign with a central echogenic line of marked hypoechoicity; type B, in which the trilinear sign and central echogenic line are not obvious; and type C, in which there is no trilinear sign and no central echogenic line, and the endometrium shows a well-delineated, homogeneous, hypoechoic lining. Type A is judged to be good, type B is moderate, and type C is poor.
(3) Uterine artery and endometrial blood flow parameters: transvaginal ultrasound was used to localize the paracervical region transversely, and Doppler spectral acquisition was carried out in selected areas with significant blood flow signals, with no less than 5 complete cardiac cycles continuously recorded at each measurement site, and the built-in analysis module of the ultrasound system was used to automatically derive the parameters related to vascular resistance, including resistance index (Resistance index, RI), pulsatility index (PI), and the ratio of peak systolic to end-diastolic blood flow velocity (S/D).
(4) Endometrial blood flow branching and typing: Applebaum’s typing method (4) was used to classify them into type I, type II, and type III. In type I, the vessels reach the subendometrium but do not enter the endometrium; in type II, the vessels reach the endometrium but not more than half of the thickness of a single layer of the endometrium; and in type III, the vessels are adjacent to or reach the midline.
(5) The direction of endometrial peristalsis: Observe endometrial peristaltic waves and record the number of waves per minute. Under resting conditions, observe endometrial fluctuations within 1 min. Endometrial peristaltic waves are classified into five types (5):

No activity (NA): The endometrium remains “static” with no movement.

Forward waves (Waves from cervix to fundus, CF): Peristaltic waves moving fromthe cervix toward the uterine fundus.

Reverse waves (Waves from fundus to cervix, FC): Peristaltic waves moving from theuterine fundus toward the cervix.

Opposing waves (OP): Simultaneous contractions originating from both the fundusand cervix.

Random waves (RA): Waves with small amplitude, lacking distinct directionality or rhythmicity.

(6) Endometrial peristaltic frequency: The algorithm for endometrial peristaltic wave frequency was that for a peristaltic wave with a fixed direction of propagation, the wave was counted once from the beginning of the wave to the end of the wave, and for a peristaltic wave with an irregular direction of propagation, the wave was counted once from the beginning of the movement to the cessation of the movement; the peristaltic frequency (waves/min) = the total number of peristaltic waves ÷ 3.

#### 2.2.4 Pregnancy outcome follow-up

All subjects received quantitative peripheral venous blood *β*-hCG testing on day 14 after embryo transfer. If the β-hCG value was lower than 2 mIU/mL, the pregnancy was determined to be non-pregnant; if the β-hCG value was higher than 2 mIU/ml, a vaginal ultrasound examination was performed by the same sonographer on day 21 post-transplantation to confirm whether the pregnancy was clinical or not. Those with a definite gestational sac detected in the uterine cavity were confirmed as clinical pregnancies, and those without were categorized as biochemical pregnancies. In this experiment, the non-pregnant and biochemical pregnancies were uniformly defined as the non-clinical pregnancy group.

### 2.3 Statistical analysis

The study was statistically analyzed using SPSS.27.0 and R language. Measures with normal distribution were expressed as (x̄ ± s), independent samples t-test was used for comparison between groups, and measures with skewed distribution were expressed as median (quartiles) [*M*(Q1, Q3)], and Mann–Whitney U-test was used for comparison between groups; counts were expressed as [*n* (%)], and X^2^ was used for comparison between groups.

Variable selection and model development were performed strictly within the training set (*n* = 354). LASSO regression was used for dimensionality reduction and feature selection of high-dimensional variables. The optimal regularization parameter (*λ*) was determined by 10-fold cross-validation, and variables with significant predictive value for RIF were screened out. The frequency of variable inclusion was calculated by 1,000 times Bootstrap resampling within the training set, and variables with frequency >50% were retained as the final predictors. A Bayesian logistic regression model was constructed based on the screened variables with an uninformative normal prior (*μ* = 0, *σ* = 2.5), and the posterior distribution was sampled by Markov chain Monte Carlo MCMC (*n* = 2000 iterations, burn-in = 1,000). Model convergence was verified by R-hat values (all < 1.01) to ensure posterior distribution stability.

To assess the robustness of performance estimates, we additionally performed 10-fold cross-validation on the training set. The model’s ability to discriminate RIF was assessed by calculating the area under the curve (AUC) from the subject’s work characteristic curve (ROC). The consistency between predicted and actual probabilities was analyzed using calibration curves to calculate the intercept (ideally 0) and slope (ideally 1). The standardized net benefit was calculated using Decision Curve Analysis (DCA) to assess the clinical decision-making value of the model over a range of different risk thresholds.

For comparison, we also trained three alternative machine learning models (Random Forest, SVM, and XGBoost) on the same training set and evaluated them on the validation set (Table 1).

**Table 1:** Comparison of model performance on the validation set.

Model	AUC	95% CI	Key advantage
Bayesian logistic regression (our model)	0.941	0.902–0.971	Interpretable ORs + credible intervals
Random Forest	0.941	0.886–0.960	Non-linear interactions
SVM (RBF kernel)	0.919	0.874–0.953	High-dimensional stability
XGBoost	0.934	0.892–0.965	Boosted tree performance

The validation set was used only once for final model evaluation, and no information from the validation set was used during feature selection or model training.

## 3 Results

### 3.1 Basic information

A total of 506 patients were enrolled in this study, including 147 cases in the RIF group and 359 cases in the CON group. All patients were divided into a training set and a validation set in a 7:3 ratio. The training set comprised 103 RIF cases and 251 CON cases. Univariate analysis revealed statistically significant differences (*p* < 0.05) between the two groups in endometrial morphology, left uterine artery S/D ratio, number of endometrial blood flow branches, endometrial blood flow classification, endometrial artery resistive index (RI), pulsatility index (PI), S/D ratio, and endometrial peristaltic frequency. The validation set included 44 RIF cases and 108 CON cases, with univariate analysis showing statistically significant differences (*p* < 0.05) in endometrial morphology, number of endometrial blood flow branches, endometrial blood flow classification, endometrial artery RI, endometrial artery PI, and endometrial peristaltic frequency between the groups. See Table 2.

**Table 2:** Comparison of baseline characteristics between the training and validation sets.

Variable	Training set (*n* = 354)		*p-value*	validation set		*P-value*
	Control (*n* = 251)	RIF (*n* = 103)		control (*n* = 108)	RIF (*n* = 44)	
General characteristics						
Age (years)	32.96 ± 2.94	33.37 ± 2.98	0.23	33.03 ± 2.76	32.52 ± 2.94	0.31
BMI (kg/m^2^)	24.32 ± 4.18	24.47 ± 4.47	0.76	23.78 ± 4.50	24.34 ± 4.15	0.48
Type of Infertility [*n* (%)]			0.56			0.21
Primary Infertility	81 (32.27)	30 (29.13)		26 (24.07)	15 (34.09)	
secondary infertility	170 (67.73)	73 (70.87)		82 (75.93)	29 (65.91)	
Duration of infertility (years)	6.13 ± 2.42	6.01 ± 2.52	0.68	5.52 ± 2.42	5.86 ± 2.20	0.42
Causes of infertility [*n* (%)]			0.62			0.57
Tubal factor	102 (40.64)	45 (43.69)		43 (39.81)	19 (43.18)	
Ovulatory dysfunction	58 (23.11)	22 (21.36)		27 (25.00)	10 (22.73)	
Male factor	71 (28.29)	28 (27.18)		31 (28.70)	12 (27.27)	
Unexplained	20 (7.97)	8 (7.77)		7 (6.48)	3 (6.82)	
Number of previous transfers	1.23 ± 0.56	1.31 ± 0.62	0.25	1.19 ± 0.51	1.27 ± 0.58	0.41
Embryo stage [*n* (%)]			0.48			0.53
Cleavage-stage	112 (44.62)	49 (47.57)		47 (43.52)	21 (47.73)	
Blastocyst	139 (55.38)	54 (52.43)		61 (56.48)	23 (52.27)	
Endocrine parameters						
AMH (ng/mL)	5.04 ± 1.02	5.07 ± 0.95	0.79	5.06 ± 1.06	4.9 ± 0.97	0.38
Basal E_2_ (pg/mL)	14.55 ± 8.32	14.65 ± 8.80	0.92	12.85 ± 6.77	11.72 ± 7.70	0.37
Basal P (ng/mL)	0.44 ± 0.25	0.47 ± 0.26	0.32	0.48 ± 0.25	0.41 ± 0.27	0.13
Basal FSH (mIU/mL)	5.15 ± 1.75	5.04 ± 1.64	0.57	4.13 ± 1.78	3.95 ± 1.76	0.56
Basal LH (mIU/mL)	4.92 ± 1.69	5.10 ± 1.69	0.36	3.79 ± 1.72	4.2 ± 1.53	0.17
Endometrial ultrasound parameters						
Endometrial thickness (cm)	1.04 ± 0.18	1.02 ± 0.20	0.35	1.03 ± 0.19	1.03 ± 0.22	0.98
Endometrial pattern [*n* (%)]			<0.05*			<0.05*
Type A	80 (31.87)	13 (12.62)		37 (34.26)	5 (11.36)	
Type B	171 (68.13)	90 (87.38)		71 (65.74)	39 (88.64)	
Type C	0 (0)	0 (0)		0 (0)	0 (0)	
Uterine artery blood flow RI left	0.81 ± 0.05	0.80 ± 0.06	0.12	0.81 ± 0.04	0.82 ± 0.06	0.28
Uterine artery blood flow RI right	0.81 ± 0.09	0.80 ± 0.06	0.27	0.81 ± 0.05	0.82 ± 0.06	0.33
Uterine artery blood flow PI Right	2.47 ± 0.89	2.54 ± 0.91	0.51	2.42 ± 0.89	2.61 ± 0.91	0.24
Uterine artery blood flow S/D Left	6.02 ± 1.96	5.47 ± 1.56	<0.05*	5.6 ± 1.43	6.11 ± 1.90	0.07
Uterine artery blood flow S/D Right	2.11 ± 1.16	2.23 ± 1.21	0.38	2.11 ± 0.33	2.23 ± 0.78	0.29
Number of endometrial blood flow branches	8.53 ± 0.84	7.10 ± 0.85	<0.05*	8.77 ± 0.90	7.14 ± 0.77	<0.05*
Endometrial blood flow type [*n* (%)]			<0.05*			<0.05*
Type I	0 (0)	0 (0)		0 (0)	0 (0)	
Type I-II	4 (1.59)	8 (7.77)		0 (0)	7 (15.91)	
Type II	247 (98.41)	94 (92.23)		108 (100)	37 (84.09)	
Endometrial artery RI	5.80 ± 2.38	6.6 ± 1.55	<0.05*	1.87 ± 1.14	2.58 ± 1.8	<0.05*
Endometrial artery PI	2.44 ± 0.85	3 ± 1.41	<0.05*	2.4 ± 0.87	3.16 ± 1.59	<0.05*
Endometrial artery S/D	2.07 ± 0.32	2.33 ± 0.76	<0.05*	5.92 ± 2.34	5.85 ± 1.86	<0.05*
Endometrial peristalsis direction [*n* (%)]			0.32			0.26
No activity (NA)	30 (11.95)	7 (6.80)		11 (10.19)	8 (18.18)	
Cervix-to-fundus (CF)	60 (23.90)	27 (26.21)		36 (33.33)	9 (20.45)	
Fundus-to-cervix (FC)	74 (29.48)	32 (31.07)		27 (25.00)	11 (25.00)	
Opposing waves (OP)	62 (24.70)	30 (29.13)		24 (22.22)	10 (22.73)	
Random waves (RA)	25 (9.96)	7 (6.80)		10 (9.26)	6 (13.64)	
Endometrial peristaltic frequency	0.55 ± 0.60	0.83 ± 0.76	<0.05*	0.44 ± 0.62	0.89 ± 0.69	<0.05*

^*^p < 0.05, statistically significant difference.

### 3.2 Variable screening and stability verification

#### 3.2.1 LASSO regression dimensionality reduction

**Figure 1:**
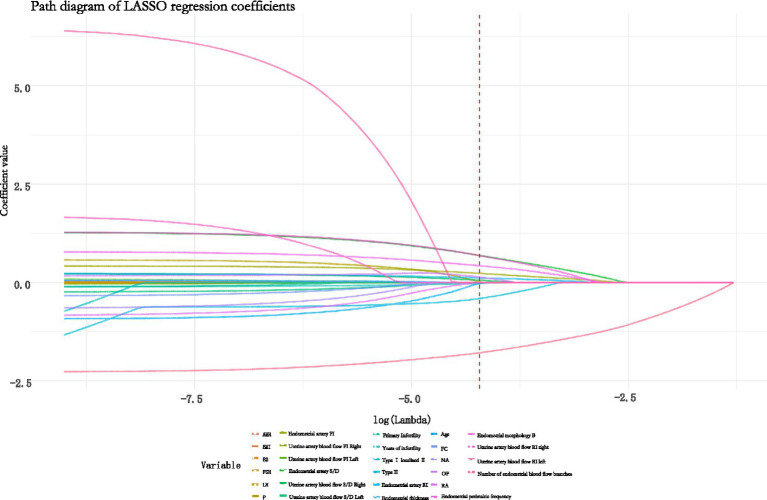
Path diagram of LASSO regression coefficients.

**Figure 2:**
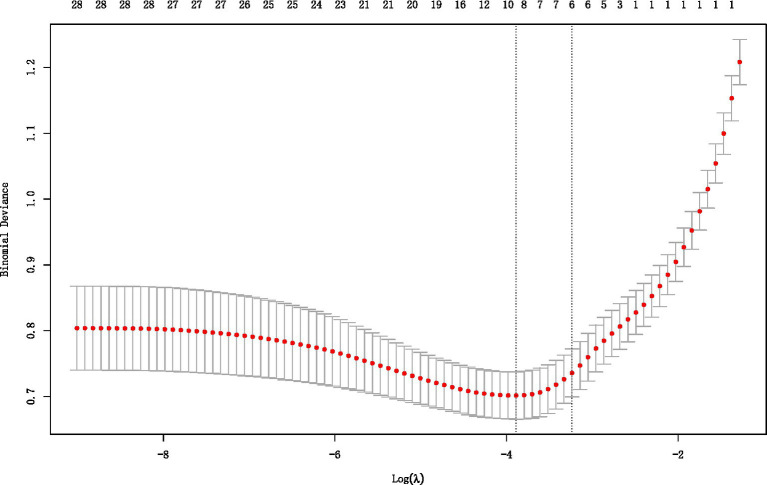
LASSO regression cross-validation results.

As shown in Figures 1, 2, LASSO regression analysis was performed to screen candidate variables and estimate coefficients within the training set. The optimal regularization parameter *λ* ≈ 0.02 (corresponding to log(λ) = −3.89) was determined through 10-fold cross-validation. This process identified variables with significant predictive value for RIF, including: endometrial blood flow branches (*β* = −1.6826), endometrial arterial RI (*β* = 0.0983), endometrial arterial PI (*β* = 0.1885), endometrial arterial S/D (*β* = 0.5706), and endometrial peristaltic frequency (*β* = 0.3577).

#### 3.2.2 Bootstrap method to verify variable stability

**Figure 3:**
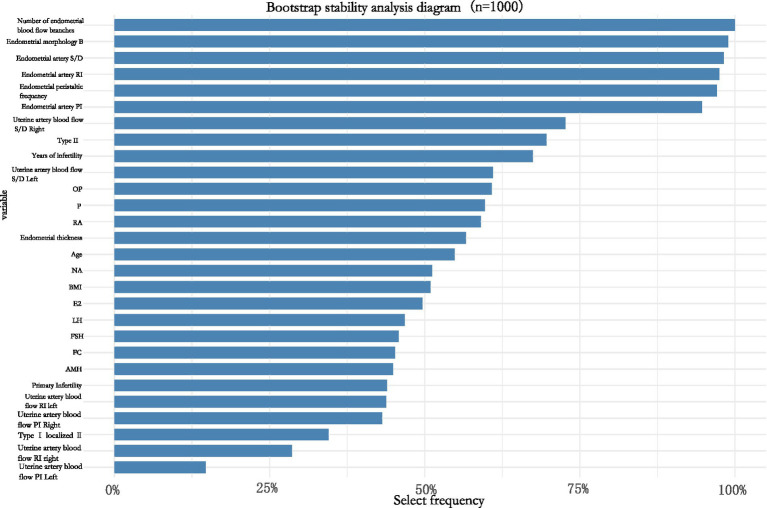
Bootstrap stability analysis diagram.

Through 1,000 iterations of Bootstrap resampling within the training set, we calculated variable selection frequencies and retained variables with frequencies exceeding 50%. As illustrated in Figure 3, the number of endometrial blood flow branches, endometrial arterial RI, endometrial arterial PI, endometrial arterial S/D, and endometrial peristaltic frequency all demonstrated selection frequencies exceeding 70%, indicating robust stability in the screening results. The validation set was not involved in any step of variable selection.

### 3.3 Bayesian model construction and convergence diagnosis

#### 3.3.1 Model parameters and *a priori* settings

A Bayesian logistic regression model with an uninformative normal prior (*μ* = 0, *σ* = 2.5) was constructed, and the posterior distribution was sampled via Markov chain Monte Carlo (MCMC) with 2000 iterations (burn-in = 1,000).

#### 3.3.2 Convergence assessment

**Figure 4:**
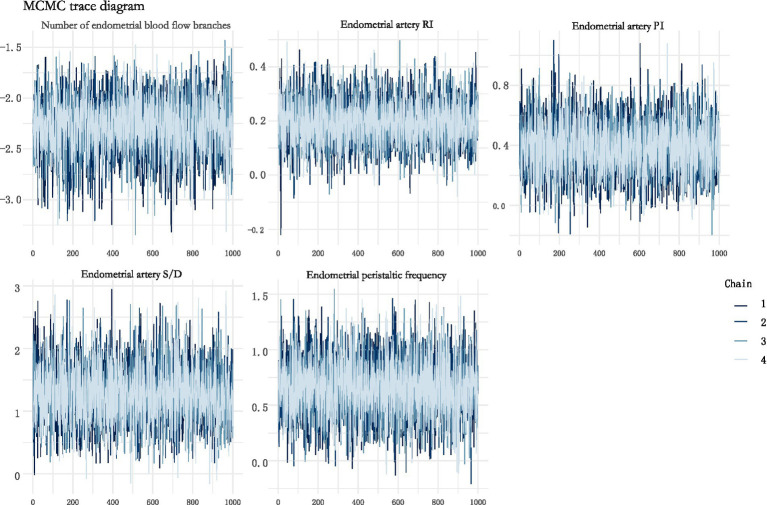
MCMC trace diagram.

As shown in Table 3, the R-hat values of all parameters were close to 1, indicating that the MCMC chain converged well. The effective sample sizes (n_eff) were all > 1,000 to satisfy the a posteriori inference requirements. The parameter trajectories (Figure 4) showed no significant periodic fluctuations or divergence, further supporting convergence.

**Table 3:** R-hat values and adequate sample size (n_eff).

Variant	R-hat value	Adequate sample size (n_eff)
Number of endometrial blood flow branches	1.000480803	3,229
Endometrial artery RI	0.999411142	4,330
Endometrial artery PI	0.999399258	4,003
Endometrial artery S/D	0.999754597	3,723
Frequency of endometrial peristalsis	0.999417748	4,700

#### 3.3.3 Posterior distribution of key variables

**Figure 5:**
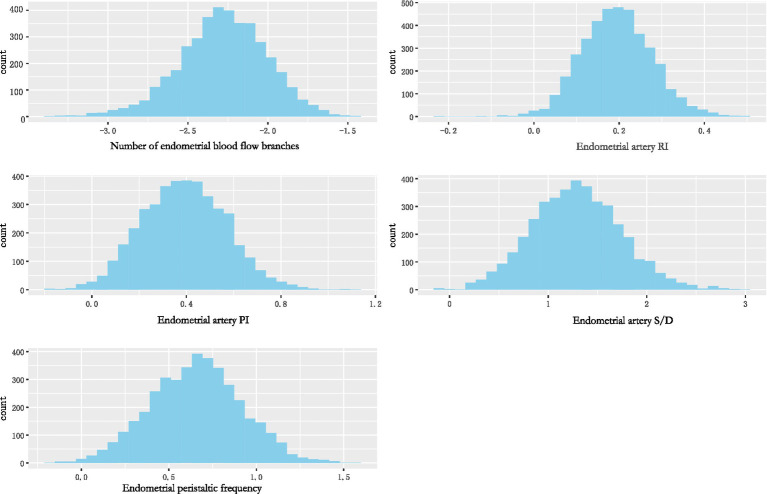
*A posteriori* distribution map.

**Figure 6:**
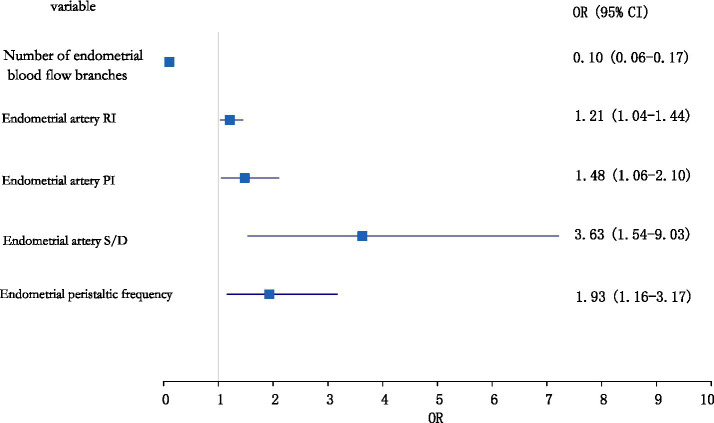
Forest map.

As shown in Figures 5, 6, increased endometrial blood flow branching (OR = 0.1, 95% CI:0.06–0.17) decreased the risk of RIF; elevated endometrial artery RI (OR = 1.21, 95% CI:1.04–1.44) increased the risk of RIF; decreased endometrial artery PI (OR = 1.48, 95% CI:1.06–2.10) was negatively associated with RIF; elevated endometrial artery S/D (OR = 3.63, 95% CI:1.54–9.03) was positively associated with RIF, and increased endometrial peristaltic frequency (OR = 1.93, 95% CI:1.931.16–3.17) increased the risk of RIF.

### 3.4 Model performance evaluation

#### 3.4.1 Ability to differentiate (AUC)

**Figure 7:**
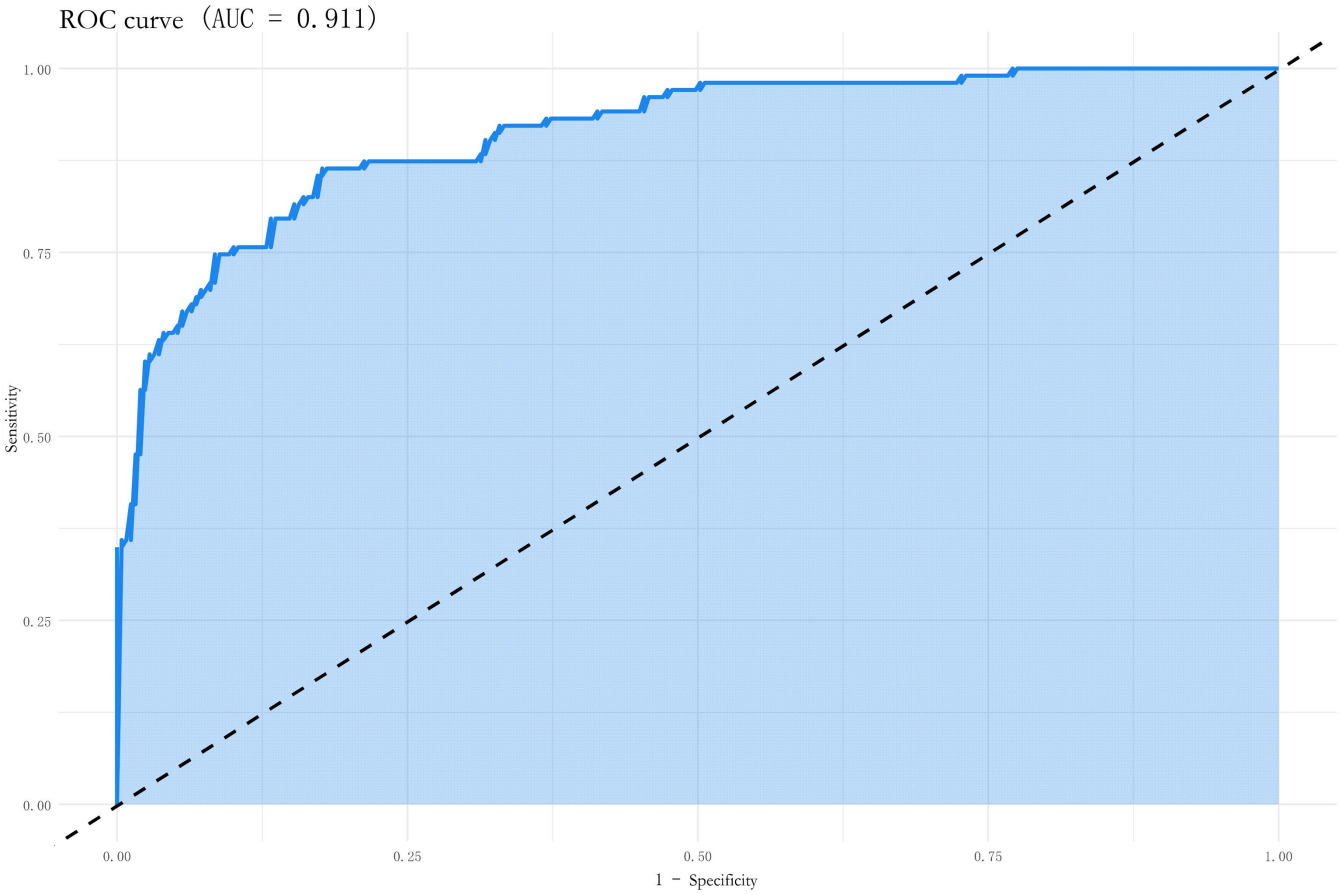
Training set ROC curve.

**Figure 8:**
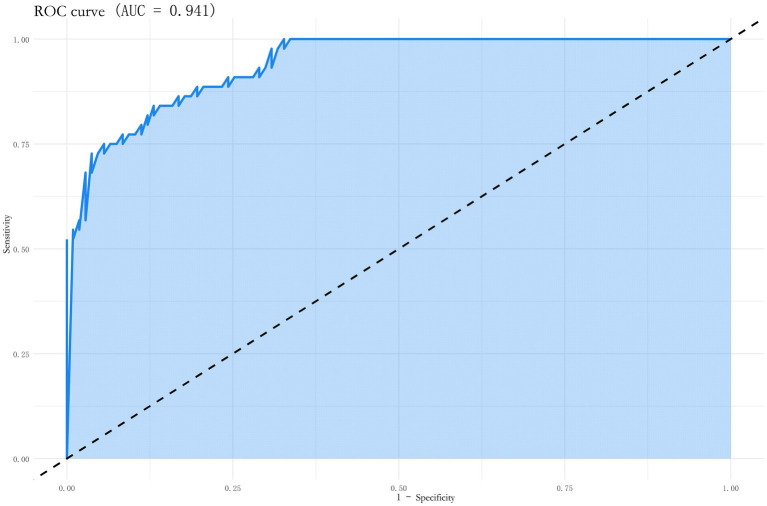
Validation set ROC curve.

As shown in Figures 7, 8, the area under the ROC curve (AUC) of the training set = 0.911; the AUC of the validation set was elevated to 0.941, and the results showed that the model had excellent discriminative ability for RIF. In addition, 10-fold cross-validation on the training set yielded a mean AUC of 0.904 ± 0.021, confirming robust discriminative performance.

We also compared our Bayesian logistic regression model with three other machine learning algorithms. On the validation set, Random Forest achieved an AUC of 0.928, SVM 0.919, and XGBoost 0.934. Our Bayesian model (AUC = 0.941) performed comparably or slightly better. More importantly, the Bayesian framework provides interpretable odds ratios and credible intervals, which are essential for clinical decision-making (Table 1).

#### 3.4.2 Calibration performance

**Figure 9:**
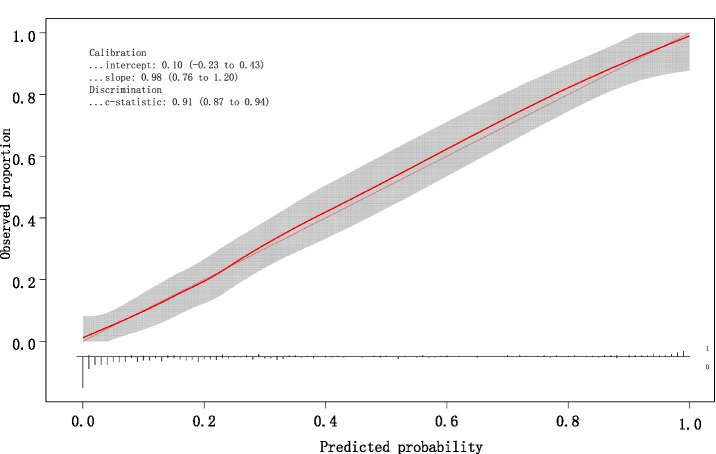
Calibration curve.

As shown in Figure 9, the calibration curve had an intercept = −0.04 and a slope = 0.98. The intercept was close to 0, and the hill was close to 1, suggesting that the predicted probabilities were highly consistent with the actual probabilities.

#### 3.4.3 Clinical relevance (decision curve analysis)

**Figure 10:**
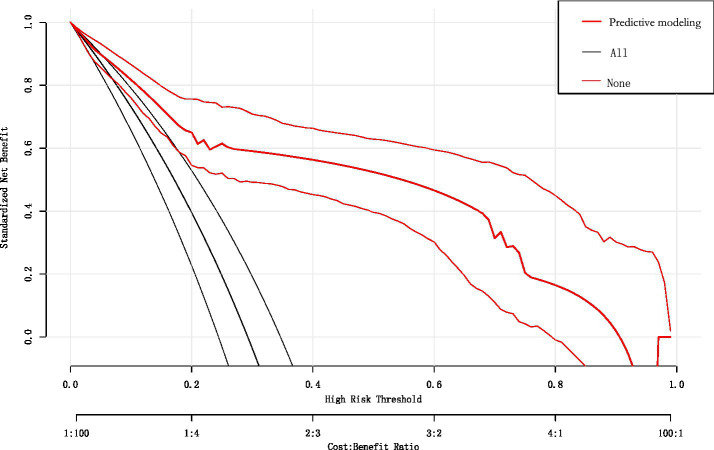
Decision curve.

As shown in Figure 10, the standardized net benefit analysis showed that the model outperformed both the “all-intervention” and “no-intervention” strategies over a range of risk thresholds from 1:100 to 100:1.

## 4 Discussion

It is clinically recognized that Endometrial receptivity is essential for embryo implantation, growth, and development and that poor Endometrial receptivity will easily affects embryo implantation and development.

### 4.1 Morphologic parameters of the endometrium

#### 4.1.1 Endometrial thickness

There is academic controversy over the clinical value of endometrial thickness as a basic morphologic index for tolerance assessment. One study showed (6) that the clinical pregnancy rate in the endometrial thickness < 8 mm group was significantly lower than that in the ≥ 8 mm group in frozen–thawed ovarian cleavage-stage embryo transfer cycles and concluded that the optimal endometrial thickness range was 8.6–15 mm and that there was no significant association between clinical pregnancy rate and endometrial thickness in this range. A Meta-analysis concluded (7) that clinical pregnancy rates, embryo implantation rates, and live birth rates in IVF/ICSI cycles were lower in the endometrial thickness <7 mm group than in the endometrial thickness ≥ 7 mm group and that those with an endometrial thickness ≥ 14 mm did not adversely affect embryo transfer outcomes. However, Coelho Neto (8) suggested that thin endometrium may be associated with poor ovarian response but is not a key predictor of clinical pregnancy. Also, there is a difference in pregnancy rate in women with thin endometrium compared to those with normal endometrium, which is more likely to be due to age and oocyte count. In the present study, there was no statistically significant difference (*p* > 0.05) in endometrial thickness between the RIF group (1.02 ± 0.20 cm) and the control group (1.04 ± 0.18 cm), which was not selected as a key variable for LASSO regression, suggesting that a comprehensive assessment in combination with other parameters is required. In this study, we excluded patients with endometrial thickness < 6 mm. Although this design limits the generalizability of our results to the thin endometrium population, it effectively controlled for the influence of this strong confounding factor. Our findings indicate that in patients with endometrial thickness ≥ 6 mm, hemodynamic and peristaltic function indicators have higher predictive value than simple thickness measurement, providing a new perspective for clinical assessment of Endometrial receptivity.

#### 4.1.2 Endometrial morphology typing

Endometrial typing is the different echo-reflections of the endometrium and adjacent myometrium. In ART treatment cycles, the most common endometrial typing prior to embryo transfer is type A. Chen (9) found within their study that type A endometrium was associated with higher clinical pregnancy rates. Some researchers, on the other hand, have concluded that endometrial typing is not associated with pregnancy outcomes. A study by Zhao noted (10) that when the endometrium was of moderate thickness (7–14 mm), there was no significant difference in clinical pregnancy rates and embryo implantation rates among patients with types A, B, and C. These inconsistencies may be due to the insufficient sample size of most studies, different study methodologies, or different time points of the study, such as some studies assessed on the day of egg retrieval. In contrast, others evaluated on the day of embryo transfer. The proportion of type A endometrium was significantly higher in the control group than in the RIF group in the present study (31.87% vs. 12.62%, *p* < 0.05). Still, it was not included in the final model, suggesting that its predictive value may be cycle-stage specific and needs to be interpreted in conjunction with dynamic monitoring.

### 4.2 Uterine artery hemodynamic parameters

The uterine artery is an important blood vessel that provides the main blood supply to the uterus and its surrounding tissues, and its blood flow status has a direct effect on the thickness of the endometrium and the level of blood perfusion. Good blood flow in the uterine arteries not only helps to maintain the stability of the endometrium but also significantly improves the rate of embryo implantation. Several studies have shown that when uterine arterial blood flow is good, the embryo receives sufficient nutrients and oxygen to promote normal development. However, if the RI or PI of the uterine artery blood flow is too high, the blood flow resistance increases and the blood perfusion decreases, which may lead to insufficient blood supply and thereby reduce the success rate of pregnancy. However, it has also been suggested that the RI and PI of uterine arteries have limitations in predicting Endometrial receptivity and pregnancy outcomes and may not serve as reliable predictors (11). In this study, there were no statistically significant differences in the RI, PI and S/D values of the left and right uterine arteries between patients with RIF and the control group. This may indicate that in patients with RIF, blood flow in the uterine arteries is not a significant cause or key factor in their embryo implantation failure.

### 4.3 Endometrial hemodynamic parameters

The endometrium is supplied with its primary nutrients by two endometrial arteries, and their perfusion levels are viewed as a key indicator for assessing Endometrial receptivity. To investigate the impact of endometrial blood supply on infertile women, several studies have evaluated the endometrium and its underlying blood supply levels in patients with unexplained infertility. The results of the studies showed that endometrial blood flow was significantly reduced in patients with unexplained infertility compared to standard controls (12). In a prospective observational study (13), the investigators enrolled a total of 165 patients who underwent their first FET cycle and assessed their endometrial blood flow perfusion levels by ultrasonography when the endometrial thickness reached 7 mm. The results of the study showed that the presence of endometrial blood flow was associated with significantly better pregnancy outcomes after embryo transfer. A previous study (14) found that endometrial blood flow RI values were considerably higher in the repeated implantation failure group than in the pregnancy group. The decrease in RI and PI, which are markers of vascular resistance, indicates good remodeling of the spiral uterine arteries and adequate blood perfusion. In addition, other studies (15) have found that lower endometrial RI and PI are positively associated with pregnancy success in fresh embryo transfer cycles. On the contrary, an elevated S/D ratio (OR = 2.06) suggests a lower end-diastolic blood flow velocity, which may reflect abnormal vasoconstrictive function or a localized hypoxic state, which may lead to the occurrence of RIF. In the present study, we found that an increase in the number of endometrial blood flow branches (OR = 0.12) was significantly associated with a decreased risk of RIF and a decrease in endometrial artery RI (OR = 0.83) and PI (OR = 0.62) were likewise negatively related to the risk of RIF, which is in agreement with the findings of previous studies, suggesting that the degree of helical arterial remodeling and the quality of endometrial microcirculatory perfusion are the central determinants of tolerability.

### 4.4 Endometrial peristalsis

The endometrium is not at rest but shows dynamic movement. Relevant studies (16) have shown that fluctuating levels of estrogen and progesterone finely regulate the contraction of the myometrium, and this contraction, in turn, triggers the endometrial lining to produce wave-like or peristaltic movements. During menstruation, the endometrium moves from the uterine fundus to the cervix, which facilitates menstrual bleeding; after estrogen increases, the direction of movement is reversed, which aids sperm transport; after ovulation, the rise in progesterone inhibits myometrial contraction, which reduces endometrial movement and facilitates embryo implantation. The presence of endometrial peristaltic waves has been demonstrated to have a significant effect on embryo implantation. Ijland (17) first documented that endometrial peristaltic frequency were significantly lower in pregnant than in nonpregnant women. At the same time, the study of Zhu L (18) further found that in fresh embryo transfer cycles, natural FET cycles, or artificial FET cycles, the frequency of uterine peristaltic waves in the nonpregnant group were significantly higher than those of the clinically pregnant group. A meta-analysis by Kuijsters (19) indicated that endometrial peristaltic frequency was negatively associated with pregnancy outcome when it was greater than 3 beats/min on the day of transfer. Excessively fast endometrial peristalsis may result in the embryo being pushed out of the uterine cavity or even into the fallopian tube, which may be detrimental to embryo implantation and may trigger an ectopic pregnancy. A similar conclusion was reached in a study by Fanchin (20), which concluded that an increase in the frequency of endometrial peristalsis prior to embryo transfer leads to a decrease in the clinical pregnancy rate and the rate of sustained pregnancies. In this study, elevated endometrial peristaltic frequency (OR = 2.42) was an independent risk factor for RIF. We, therefore, hypothesized that frequent localized endometrial peristalsis may be an unfavorable factor contributing to impaired embryo implantation in patients with RIF.

### 4.5 Predictive modeling of Endometrial receptivity for repeated implantation failure

In this study, the L1 regularization constraint was introduced by LASSO regression to screen five key variables (the number of endometrial blood flow branches, endometrial arterial RI, endometrial arterial PI, endometrial arterial S/D, and endometrial peristaltic frequency) from eight candidate indicators, which avoided the subjective bias and overfitting risk of the traditional stepwise regression method. Second, the Bayesian model quantified the posterior distributions of the parameters through Markov chain Monte Carlo (MCMC) sampling, which not only provided point estimates of the effects of the variables (e.g., OR values) but also specified the range of their uncertainty (95% confidence intervals). This uncertainty quantification is a key advantage over conventional logistic regression or “black-box” machine learning models, as it allows clinicians to gauge confidence in each prediction.

From a clinical practice perspective, the present model has a dual value: first, by integrating hemodynamic and peristaltic features, it provides a new perspective on the mechanistic resolution of RIF. For example, decreased RI and PI reflect good spiral artery remodeling, suggesting that improving endometrial perfusion (e.g., using low-dose aspirin or vitamin E) may be a target for intervention. In contrast, increased peristaltic frequency supports the individualized application of contraction inhibitors (e.g., atosiban). Second, the model’s high discriminatory power (AUC = 0.884) and calibration performance (slope ≈ 1) allow it to be embedded in clinical decision-making systems to assist physicians in identifying high-risk patients and optimizing transplant timing. For example, intensive strategies such as uterine perfusion can be prioritized and recommended for high-risk patients with a predicted probability of > 80% of RIF. Future studies can further incorporate molecular markers (e.g., microRNA or metabolomics data), construct multi-omics prediction models, and promote RIF diagnosis and treatment from empirical guidance to precision medicine. Future prospective multicenter studies are needed to explore the dynamic molecular regulatory network of Endometrial receptivity.

In this study, the key role of endometrial hemodynamics (the number of endometrial blood flow branches, endometrial arterial RI, endometrial arterial PI, endometrial arterial S/D) and endometrial peristaltic frequency in patients with RIF was systematically revealed for the first time by LASSO regression and Bayesian modeling. The constructed predictive model had excellent discriminatory ability and clinical utility, providing a quantitative tool for precise intervention in RIF. Future studies should explore the dynamic molecular regulatory network of Endometrial receptivity.

Recent studies have demonstrated the power of multi-modal data fusion and advanced machine learning in clinical prediction (21). While our model relies on ultrasound parameters only, future studies can further incorporate molecular markers (e.g., microRNA or metabolomics data) to construct multi-omics prediction models, as suggested by emerging research (22).

We acknowledge that a single train-validation split may be a limitation. However, our additional 10-fold cross-validation produced consistent results (mean AUC 0.904), and external validation in a prospective multicenter cohort is needed to further confirm generalizability.

Moreover, although our Bayesian logistic regression model performed similarly to other machine learning methods (RF, SVM, XGBoost), its superior interpretability and uncertainty quantification make it more suitable for clinical practice.

Future prospective multicenter studies are needed to explore the dynamic molecular regulatory network of Endometrial receptivity.

## 5 Conclusion

This study provides a clinically applicable predictive model for RIF, highlighting the critical role of endometrial hemodynamic and peristaltic characteristics. The Bayesian framework offers uncertainty estimates that can guide personalized interventions. These findings provide new targets for precision diagnosis and treatment.

## Data Availability

The datasets used in this study are available from the corresponding author upon reasonable request.
